# Fate of the Dermal Component of Micrografts in Full-Thickness Wounds

**Published:** 2014-10-09

**Authors:** Mansher Singh, Kristo Nuutila, Carla Kruse, Edward J Caterson, Scott R. Granter, Elof Eriksson

**Affiliations:** ^a^Division of Plastic and Reconstructive Surgery; ^b^Department of Pathology, Brigham and Women's Hospital, Boston, Mass

**Keywords:** micrografts, full-thickness wound, wound healing, split-thickness skin graft, mincing

Dear Sir,

The most widely used skin graft in the treatment of full-thickness wounds, caused by major burns or trauma, is split-thickness skin grafts (STSGs).^1^^-^3 In an attempt to increase the expansion ratio of the STSGs, our laboratory has previously shown that autologous minced skin grafting accelerates reepitheliazation.^4^^-^7 The micrografts (0.8 mm × 0.8 mm) are generated by controlled mincing of STSGs and enables early wound coverage of full-thickness wounds (SteadMed Inc, Fort Worth, Texas).^8^^-^11 The fate of the dermal component of the micrografts, which is relatively unknown, was objectively evaluated in this study.

All animal procedures were approved by the Harvard Medical Area Standing Committee on Animals and have been described in previous reports.^4^^-^7 Briefly, full-thickness wounds were created on 4 female Yorkshire pigs and wound healing was studied by application of micrografts and histological study of wound biopsies on the days 6, 10, and 14. Sixteen slides were evaluated in each group and dermal histologic findings were divided in 3 categories as follows: (A) Incorporated—The dermal components of micrograft were completely interior to the neoepidermis of the wound. (B) Expelled through transepidermal elimination—The dermal components of the micrograft were completely exterior to the neoepidermis of the wound. (C) Indeterminate—Micrograft could not be classified as incorporated or expelled, based on histology (Fig 1). We also studied the fate of the stratum corneum and residual epidermis of the micrografts during histological review.

The mean number of visible micrografts per slide was similar for day 6 (3.06) and day 10 (2.93). Even though it was not significantly higher than day 14 (1.62), there was a trend toward significance (*P* = .06 for day 6 vs day 14 and *P* = .07 for day 10 vs day 14) (Fig 2a). The dermis of almost 90% of the visible micrografts were incorporated in the neodermis on day 6 and day 10 with less than 10% transepidermal elimination of the dermis of the micrografts in each group (Fig 2b). Early incorporation into the neodermis and the continued migration of the micrograft toward and beyond neoepidermis would explain the relatively skewed ratio of incorporated (69%) and expelled micrografts (15%) on day 14 (Fig 2b).

Since most of the micrografts had migrated to the neoepidermis by day10, it appears to be an optimal time point to evaluate the outcome of micrografts. The stratum corneum, residual epidermis, and the dermal components of the micrografts seem to have completely different outcomes. The stratum corneum of the micrograft was uniformly extruded through the neoepidermis while the rest of the epidermis was uniformly incorporated into the neoepidermis. Based on our findings, the dermis of approximately 90% of the visible micrografts actively contributes to the formation of neodermis in full-thickness wounds while less than 10% get expelled through the neoepidermis.

## Figures and Tables

**Figure 1:**
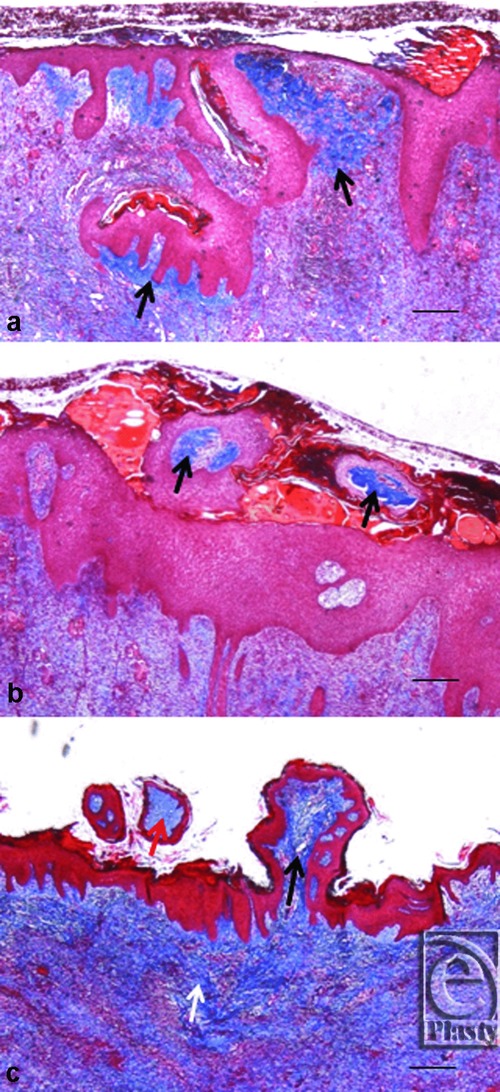
Masson's Trichrome staining demonstrating possible outcomes of skin micrograft dermis. (*a*) Incorporated—The dermal components of micrograft (arrow) are completely interior to the neoepidermis of the wound. (*b*) Expelled through transepidermal elimination—The dermal components of the micrograft (arrow) are completely exterior to the neoepidermis of the wound. (*c*) Indeterminate—Micrograft (black arrow) cannot be classified as incorporated or expelled, as compared to incorporated (white arrow) or transepidermaly expelled dermis (red arrow), based on histology. Scale represents 500 μm.

**Figure 2:**
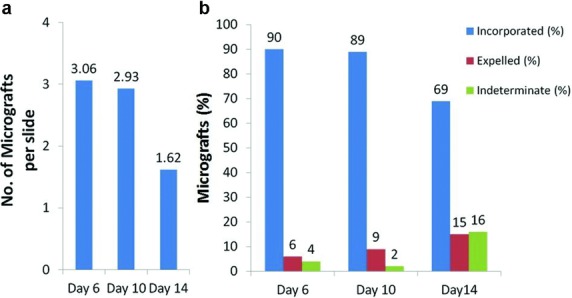
Micrografts on day 6, day 10, and day 14. (*a*) Mean number of visible micrografts per slide. (*b*) Micrograft dermis expressed as a percentage of the total number of visible micrografts, categorized as Incorporated, Expelled through transepidermal elimination, or Indeterminate.

## References

[1] Gibran NS, Boyce S, Greenhalgh DG (2007). Cutaneous wound healing. J Burn Care Res.

[2] Brusselaers N, Pirayesh A, Hoekema H (2010). Skin replacement in burn wounds. J Trauma.

[3] Tanner JC, Vandeput J, Olley JF (1964). The mesh skin graft. Plast Reconstr Surg.

[4] Svensjö T, Pomahac B, Yao F, Slama J, Wasif N, Eriksson E (2002). Autologous skin transplantation: comparison of minced skin to other techniques. J Surg Res.

[5] Hackl F, Bergmann J, Granter SR (2012). Epidermal regeneration by micrograft transplantation with immediate 100-fold expansion. Plast Reconstr Surg.

[6] Hackl F, Kiwanuka E, Philip J (2014). Moist dressing coverage supports proliferation and migration of transplanted skin micrografts in full-thickness porcine wounds. Burns.

[7] Kiwanuka E, Hackl F, Philip J, Caterson EJ, Junker JP, Eriksson E (2011). Comparison of healing parameters in porcine full-thickness wounds transplanted with skin micrografts, split-thickness skin grafts, and cultured keratinocytes. J Am Coll Surg.

[8] Boggio P, Tiberio R, Gattoni M, Colombo E, Leigheb G (2008). Is there an easier way to autograft skin in chronic leg ulcers? ‘Minced micrografts’, a new technique. J Eur Acad Dermatol Venereol..

[9] Danks RR, Lairet K (2010). Innovations in caring for a large burn in the Iraq war zone. J Burn Care Res.

[10] Pertusi G, Tiberio R, Graziola F, Boggio P, Colombo E, Bozzo C (2012). Selective release of cytokines, chemokines, and growth factors by minced skin in vitro supports the effectiveness of autologous minced micrografts technique for chronic ulcer repair. Wound Repair Regen.

[11] Emsem IM (2008). The use of micrografts and minigrafts together with advancement of temporalis fascia and its periosteum on the treatment of burn alopecia. J Craniofac Surg.

